# Congenital Hypogonadotropic Hypogonadism Due to GNRH Receptor Mutations in Three Brothers Reveal Sites Affecting Conformation and Coupling

**DOI:** 10.1371/journal.pone.0038456

**Published:** 2012-06-05

**Authors:** Javier A. Tello, Claire L. Newton, Jerome Bouligand, Anne Guiochon-Mantel, Robert P. Millar, Jacques Young

**Affiliations:** 1 Centre for Integrative Physiology, School of Biomedical Sciences, University of Edinburgh, Edinburgh, United Kingdom; 2 University of Cape Town/Medical Research Council Receptor Biology Unit, University of Cape Town, Cape Town, South Africa; 3 Univ Paris-Sud, Faculté de Médecine Paris-Sud UMR-S693, Le Kremlin Bicêtre, France; 4 INSERM U693, IFR93, Le Kremlin-Bicêtre, France; 5 Assistance Publique-Hôpitaux de Paris, Hôpital Bicêtre, Service de Génétique Moléculaire, Pharmacogénétique et Hormonologie, Le Kremlin Bicêtre, France; 6 Mammal Research Institute, University of Pretoria, Pretoria, South Africa; 7 Service d’Endocrinologie et des Maladies de la Reproduction, Le Kremlin Bicêtre, France; University of Iowa, United States of America

## Abstract

Congenital hypogonadotropic hypogonadism (CHH) is characterized by low gonadotropins and failure to progress normally through puberty. Mutations in the gene encoding the GnRH receptor (*GNRHR1*) result in CHH when present as compound heterozygous or homozygous inactivating mutations. This study identifies and characterizes the properties of two novel *GNRHR1* mutations in a family in which three brothers display normosmic CHH while their sister was unaffected. Molecular analysis in the proband and the affected brothers revealed two novel non-synonymous missense *GNRHR1* mutations, present in a compound heterozygous state, whereas their unaffected parents possessed only one inactivating mutation, demonstrating the autosomal recessive transmission in this kindred and excluding X-linked inheritance equivocally suggested by the initial pedigree analysis. The first mutation at c.845 C>G introduces an Arg substitution for the conserved Pro 282 in transmembrane domain (TMD) 6. The Pro282Arg mutant is unable to bind radiolabeled GnRH analogue. As this conserved residue is important in receptor conformation, it is likely that the mutation perturbs the binding pocket and affects trafficking to the cell surface. The second mutation at c.968 A>G introduces a Cys substitution for Tyr 323 in the functionally crucial N/DPxxY motif in TMD 7. The Tyr323Cys mutant has an increased GnRH binding affinity but reduced receptor expression at the plasma membrane and impaired G protein-coupling. Inositol phosphate accumulation assays demonstrated absent and impaired Gα_q/11_ signal transduction by Pro282Arg and Tyr323Cys mutants, respectively. Pretreatment with the membrane permeant GnRHR antagonist NBI-42902, which rescues cell surface expression of many *GNRHR1* mutants, significantly increased the levels of radioligand binding and intracellular signaling of the Tyr323Cys mutant but not Pro282Arg. Immunocytochemistry confirmed that both mutants are present on the cell membrane albeit at low levels. Together these molecular deficiencies of the two novel *GNRHR1* mutations lead to the CHH phenotype when present as a compound heterozygote.

## Introduction

The dynamic secretion of synchronized gonadotropin releasing hormone (GnRH) pulses from discrete nerve terminals into the hypophyseal portal blood is the ultimate regulatory signal of the brain to control reproduction [1]. The GnRH receptor, present on pituitary gonadotropes, acts to link the hypothalamus and gonads by binding GnRH and transducing this signal intracellularly to regulate the synthesis and secretion of the gonadotropins, luteinizing hormone (LH) and follicle-stimulating hormone (FSH) [2]. Dysfunction of this ligand/receptor pair disrupts gonadotropin secretion and ultimately leads to insufficient gonadal maturation, presenting in patients as non-syndromic and normosmic (with normal sense of smell) congenital hypogonadotropic hypogonadism (CHH) [3], [4].

Twenty two natural inactivating mutations of the human GNRHR have been identified in patients with CHH and can be classified as either partial or complete loss-of-function mutations [4]. Partial loss-of-function mutants may have one or more of the following traits: reduced ligand binding affinity, decreased intracellular signaling capacity and decreased receptor expression at the cell surface due to altered trafficking of nascent receptor protein. Interestingly, many trafficking deficient mutants can be functionally rescued following pretreatment with cell permeant non-peptidic GnRH antagonists (termed pharmacoperones), which assist newly synthesized GnRHR protein to fold correctly within the endoplasmic reticulum (ER) and be efficiently targeted to the plasma membrane [5].

In the present study, we characterize two novel *GNRHR1* missense mutations Pro282Arg and Tyr323Cys identified in patients with normosmic CHH (nCHH), which masqueraded as an X-linked deficiency. Measurement of membrane binding, Gα_q/11_ protein signaling as well as the ability to rescue nascent receptor trafficking show that Pro282Arg is a complete loss-of-function mutant and Tyr323Cys is a partial loss-of-function mutant. The presence of CHH in our compound heterozygote patients suggests that the poor trafficking and compromised signaling of the Tyr323Cys mutant is unable to meet the necessary molecular demands in the gonadotrope cell to compensate for the Pro282Arg attributed complete loss-of-function *in vivo*.

## Materials and Methods

### Ethics Statement

All the participants gave their written informed consent for hormonal exploration and genetic analyses, in keeping with the provisions of the French Bioethics Law and the Declaration of Helsinki and after approval by the Bicetre Hospital ethic committee (Comite de protection des personnes Ile de France, Hopital Bicetre).

### Hormonal Assays

We measured serum levels of LH, FSH, inhibin B, testosterone and estradiol (E2) levels with immunoradiometric assay, enzyme-linked immunosorbent assay, or radioimmunoassay (RIA) as previously reported [6]–[8]. Briefly, the detection limits of the LH and FSH assays were 0.15 IU/L and 0.2 IU/L, respectively. The intra and inter-assay coefficients of variation (CVs) were, 1.5% and 5.2% for LH, 2.7% and 5.5% for FSH, respectively.

Serum total testosterone (TT) was measured with a direct RIA on an Orion Diagnostica device (Spectria, Espoo, Finland) with a detection limit of 0.02 ng/mL (0.06 nmol/L). The intra-assay and inter-assay CVs were 3.8% and 4.8% at 3.3 ng/mL and 2.6 ng/mL (11.4 and 9.1 nmol/L), respectively, and the intra-assay and inter-assay CVs were 7.5% and 7.0% at 0.46 ng/mL and 0.35 ng/mL (1.6 and 1.2 nmol/L), respectively, for TT.

Serum total E2 was measured with a sensitive direct RIA on an Orion Diagnostica device (Spectria). The detection limit was 2 pg/mL (7.3 pmol/L). The intra-assay and the inter-assay CVs were 2.8% and 5.8% at 23.5 pg/mL and 25.4 pg/mL (87 pmol/L and 94 pmol/L), respectively, and the intra-assay and inter-assay CVs were 18.1% and 17.6% at 4.6 pg/mL and 3.3 pg/mL (17 pmol/L and 12 pmol/L), respectively, for serum total E2.

### Reagents

NBI-42902 (1-(2,6-difluorobenzyl)-3-[(*2R*)-amino-2-phenethyl]-5-(2-fluoro-3-methoxyphenyl)-6-methyluracil) was generously provided by Neurocrine Biosciences (San Diego, CA). His^5^-D-Tyr^6^-GnRH1 was iodinated in house as previously described [9]. All cell culture reagents were obtained from Sigma-Aldrich (Dorset, UK) or Invitrogen (Life technologies, Paisley, UK) unless otherwise stated.

### DNA Constructs Design

The wild type human GNRHR cDNA was modified to encode an amino-terminal Hemagglutinin (HA) tag and cloned into pcDNA3.1(+) (Invitrogen). The two *GNRHR1* missense mutations were prepared using a Phusion Site-Directed Mutagenesis Kit (Finnzymes, Vantaa, Finland). Phosphorylated (5′-end) sense and antisense primers were as follows: (p.Pro282Arg; c.845 C>G) Sense: 5′- CTGCTGGACT**CGC**TACTACGTACT-3′; Antisense: 5′-ACAGTAAAACTAGTGGCAAATGCAA-3′; (Y323C; c.968 A>G) Sense: 5′- TCCATTAATC**TGT**GGATATTTTTCTCTG-3′; Antisense: 5′-TCAAAGCATGGGTTTAAAAAGG-3′. Expression construct sequences were confirmed with Sanger sequencing and receptor protein expression was verified by immunocytochemistry.

### Cell Culture, Transfection and Rescue Treatment

Wild type or mutant *GNRHR1* constructs were transiently transfected into COS-7 (ATCC CRL-1651) cells by transfection using electroporation with a Gene Pulser (Bio-Rad Laboratories, Hertfordshire, UK) at 230 V, 950 µF with 15 µg of DNA/0.4 cm cuvette (1.5×10^7^ cells; 0.7 mL). To assess rescue of receptor trafficking, either NBI-42902 (1 µM) or DMSO (vehicle control) was added immediately before plating into 24-well plates. Cells were grown for 28 h in Complete Medium (Dulbecco’s modified Eagle’s Medium (DMEM) supplemented with 10% fetal bovine serum, antibiotics and 2 mM L-glutamine) and then washed (4×30 min each) with Wash Medium (HEPES-DMEM supplemented with 2% DMSO, 0.1% bovine serum albumin and penicillin/streptomycin). The Wash Medium was removed and the cells were further incubated with the appropriate medium (as mentioned below) overnight (∼16 h) and then washed again before the radioligand binding and IP accumulation assays were performed.

### Whole Cell Competitive Radioligand Binding Assays

Radioligand binding assays were performed on intact COS-7 48 h after transfection following the treatment with or without 1 µM NBI-42902. After the first round of washing (28 h post-transfection), Complete Medium was added to cells and further grown overnight. In the morning, the cells were washed again and intact cells were incubated with radiolabeled GnRH analog ([^125^I]-His^5^-D-Tyr^6^-GnRH1, 100,000 cpm/well) and graded concentrations of unlabeled GnRH1, prepared in Binding Buffer (HEPES-DMEM supplemented with 0.1% BSA), for 4 h at 4°C. After incubation, free radioligand was removed from the cells by two rapid washes with ice-cold PBS and the cells were solubilized with NaOH (0.1 M). The radioactivity was determined using a Wizard™ 1470 automatic gamma counter (Perkin Elmer).

### Inositol Phosphate Accumulation Assays

Measurements of GnRH-elicited IP accumulation were performed in COS-7 cells 48 h after transfection following incubation with or without 1 µM NBI-42902. After the first round of washing at 28 h, Labeling Medium (inositol-free DMEM supplemented with 1% dialyzed heat-inactivated fetal calf serum, antibiotics and 2 mM L-glutamine with 0.5 µCi/well of myo-[2-^3^H]Inositol [Perkin Elmer, Buckinghamshire, UK]) was added and the cells were further grown overnight. In the morning, the cells were washed again and incubated with IP Medium (HEPES-DMEM supplemented with 0.1% bovine albumin and 10 mM LiCl) for 30 min at 37°C and then stimulated with graded concentrations of GnRH1 in IP Medium for 1 h at 37°C. Reactions were terminated by removal of ligands and the cells were lysed with 200 µl of ice-cold formic acid (0.1 M). Quantification of total ^3^H-labeled inositol phosphates from cellular extracts was performed by the multiwell filtration method [10].

### Confocal Microscopy

COS-7 cells were electroporated (as described previously), diluted in Complete Medium, seeded on poly-D-lysine coated glass coverslips and grown for 48 h at 37°C. Cells were then washed with ice-cold PBS and fixed with 4% paraformaldehyde for 10 min at room temperature, washed 3x with PBS, permeabilized with 0.3% Triton X-100, washed 1x with PBS, and blocked with Blocking Solution (PBS containing 10% normal donkey serum and 0.5% NEN block [Perkin Elmer, UK]) for 45 min at room temperature. The cells were then incubated with rabbit anti-HA primary antibody (ab9110, 1∶5000, Abcam UK) for 2 h at 37°C and then Alexa Fluor® 488 conjugated donkey anti-rabbit secondary antibody (Invitrogen, 1∶400) for 1 h at 37°C. Cells were counterstained with Hoechst 33342 (10 µg/ml) for 10 min at room temperature and visualized using a Zeiss LSM510 Meta inverted confocal laser scanning microscope (Carl Zeiss, Herts, UK) equipped with a (DAPI/FITC/Rhod) filter set and a 63x oil objective lens (Zeiss). The exposure settings were determined empirically for each channel from control background and unchanged within an experiment. Images were acquired at 1024×1024 pixel resolution and using 0.3 µm Z-stacks. Image stacks were imported into Image J software where average intensity images were produced after which scale bars were embedded and files were saved in tiff format.

### Presentation of Data and Statistics

For analyses of competitive radioligand binding and inositol phosphate accumulation, data representing the mean ± SEM from at least three independent experiments (in which each data point was measured in triplicate) were plotted using a one-site model of binding using Prism 5.0 (GraphPad Software, San Diego, CA). The data from mutant receptors and treatment with NBI-42902 were expressed relative to a wild type control included in each transfection. Sigmoidal dose-reponse curves were fitted to the relevant data sets using non-linear regression and IC_50_, *B*_max_, EC_50_, and *E*_max_ values were determined. The effect of NBI-42902 treatment on each receptor was analyzed for statistical significance by Student’s *t* test and indicated by an asterisk in the tables (*P*≤0.05).

## Results

### Clinical Evaluation

**Figure 1:**
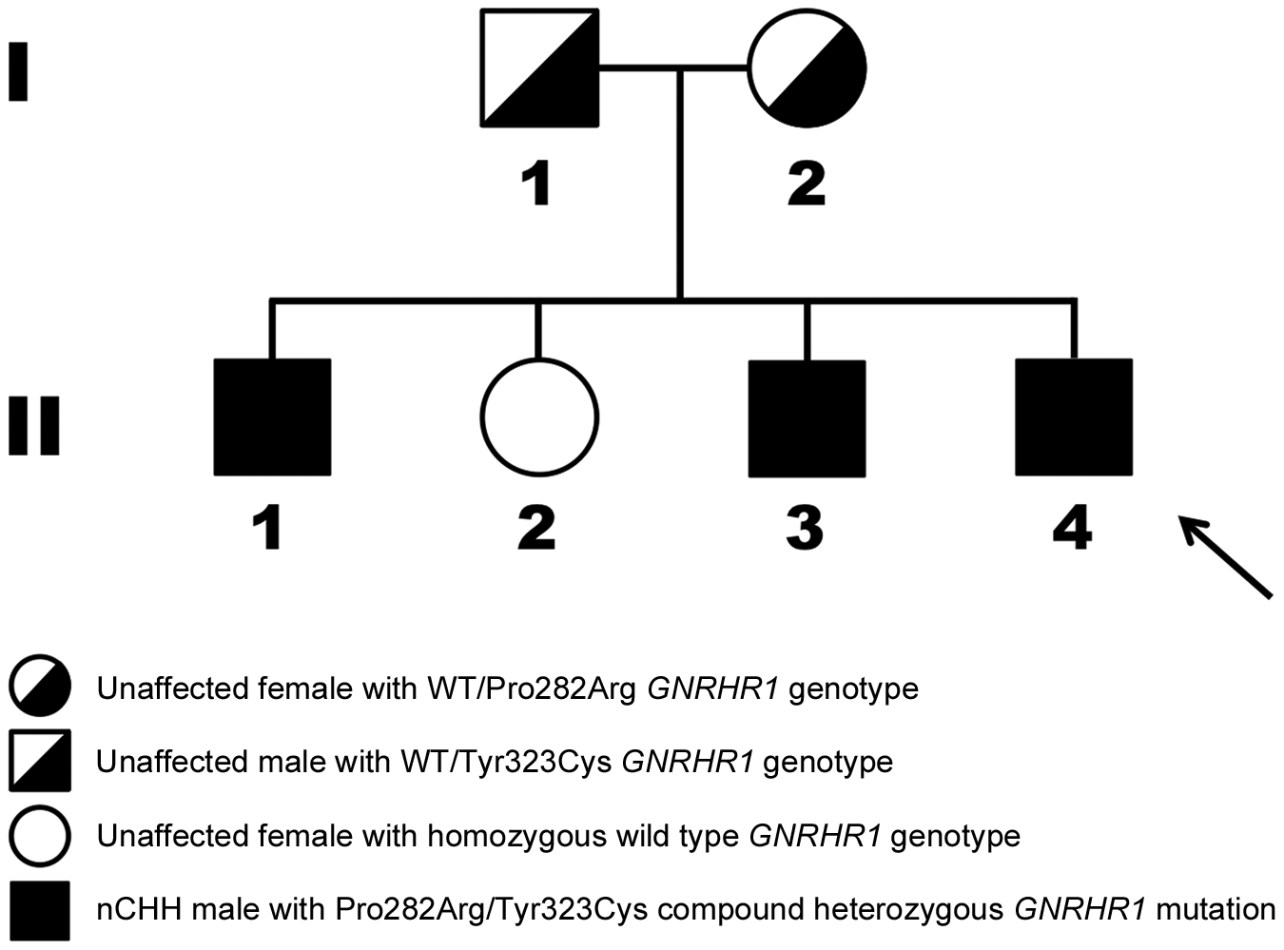
Pedigree showing two heterozygous mutations (Pro282Arg and Tyr323Cys) in the gene encoding *hGNRHR1* (NM_000406.2; NP_000397.1). A C-to-G transversion at cDNA nucleotide 845 (c.845C>G) and a A-to-G transition at cDNA nucleotide position 968 (c.968A>G) leading to a substitution of Proline at residue 282 for Arginine (p.282P>R) and a substitution of Tyrosine at residue 323 for Cysteine (p.323Y>C). Upper panel: Heterozygous Tyr323Cys and wild type allele in unaffected father (I-1); Heterozygous Pro282Arg and wild type allele in unaffected mother (I-2); Bottom panel: Homozygous wild type alleles in an unaffected sister (II-2); Compound heterozygous Pro282Arg and Tyr323Cys brothers (II-1, II-3) and proband (II-4 arrowed).

The proband (subject II-4, Fig. 1) was a Caucasian French man born to non-consanguineous eugonadal French parents. He was referred to the clinic at 18 years of age for absent pubertal development. Physical examination showed typical signs of hypogonadism, with small intrascrotal testes (2 mL). He had very low levels of plasma testosterone, serum LH and FSH and displayed a blunted response to GnRH challenge (Table 1). His height was 183 cm and his weight was 65 kg. He had a normal (46 XY) karyotype.

**Table 1:** Clinical and hormonal characteristics of family members.

Subject (see Fig. 1) (*GNRHR1* genotype)	I-1 (Tyr323Cys/WT)	I-2 (Pro282Arg/WT)	II-1 (Tyr323Cys/Pro282Arg)	II-2 (WT/WT)	II-3 (Tyr323Cys/Pro282Arg)	II-4 (Tyr323Cys/Pro282Arg)
Sex/age at initial evaluation (years)	M/47	F/44	M/21	F/34	M/20	M/18
Spontaneous puberty	yes	yes	no	yes	no	no
Clinical examination^a^	Eugonadic; Penis: 14 cm length; TV:19 mL	Eugonadic; Spontaneousregular menses;4 Spontaneous pregnancies	Hypogonadic; Penis:4 cm length; TV:2 mL, Intrascrotal	Eugonadic; Spontaneousregular menses;4 Spontaneous pregnancies	Hypogonadic; Penis:6 cm length; TV:3 mL, Intrascrotal	Hypogonadic; Penis:3 cm length; TV:2 mL, Intrascrotal
Sense of smell (olfactometry)	–	–	normal	–	normal	normal
Ta,b (ng/mL)/E2 (pg/mL)	5.7/−	−/47	0.2/−	−/62	0.4/−	0.15/−
LHa,b (IU ; basal/stimulated^c^)	3.8	3.8	0.3/0.6	5.7/−	0.7/−	0.5/0.8
FSHa,b (IU ; basal/stimulated^c^)	4.3	3.9	0.9/0.8	4.4/−	0.9/−	0.8/1.1
Inhibin B	186	–	33	–	26	39
Other pituitary functions^d^	–	–	normal	–	normal	normal
Pituitary and olfactory bulbs MRI	–	–	normal	–	normal	normal

WT: wild type *GNRHR1*; TV: mean testicular volume (normal range in post pubertal men: 12–30 mL). MRI: magnetic resonance imaging.

a At diagnosis before therapy in affected patients.

b Normal range in adults, basal LH: 2.8–7.1 IU/L; basal FSH: 2.4–7.0 IU/L, in post menopausal women: LH: 14–132 IU/L, FSH: 28–171; T in men: 2.8–9.0 ng/mL, Inhibin B in men: 98–366); E2 in women: 24–90 pg/mL (early follicular phase).

c GnRH: 100 µg intravenous.

d Normal cortisol and GH under hypoglycemia challenge test. Normal free T4 and TSH, normal prolactin.

- : Not done.

The proband’s two older affected brothers (subjects II-1 and Subject II-3, Fig. 1) were also referred to the clinic for absent pubertal development at 21 and 20 years of age, respectively. They both presented with typical aspects of hypogonadism in which the diagnosis of congenital hypogonadotropic hypogonadism was made given the concomitant very low serum testosterone, low gonadotropin levels and the blunted gonadotropin response (LH and FSH) after GnRH stimulation (Table 1).

All the male patients studied here had normal pituitary glands and olfactory bulbs when visualized using magnetic resonance imaging (MRI) and had a normal sense of smell when assessed with olfactometry. They all had no renal or craniofacial abnormalities and had normal circulating iron, ferritin, and prolactin levels. Their adrenal and thyroid function was normal and had otherwise normal pituitary function. None had any other phenotypic abnormality.

### Molecular Analysis

The proband was screened for mutations in genes known to be associated with CHH including *GNRH1*, *GNRHR1, KISS1*, *KISS1R*, *TAC3* and *TACR3* and those known to be associated with Kallmann syndrome *FGFR1*, *PROK2* and *PROK2R*. Analysis of these genes were performed as previously reported [6], [11]–[14]. In addition, two genes responsible for the X-linked forms of Kallmann syndrome and CHH associated with adrenal hypoplasia congenita (*KAL1* and *NR0B1*, respectively) were also screened as previously reported [13], [15].

**Figure 2:**
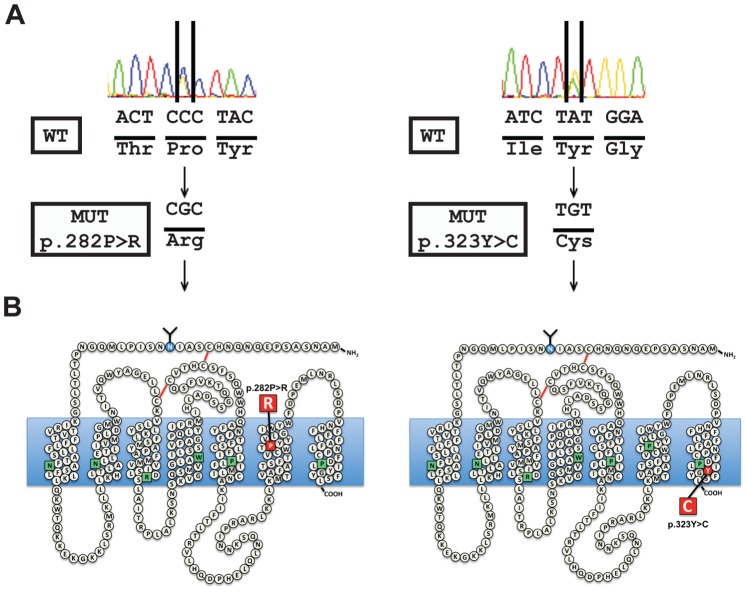
DNA sequence traces and associated amino acid changes in the *hGNRHR1* gene of the propositus. A, Forward sequence traces of the propositus encompassing the heterozygotic mutations in *GNRHR*, c.968A>G and c.968A>G. Nucleotides changes are indicated by arrows. MUT, Mutant; WT, wild type. B, The resultant amino acid changes are presented for the mutants compared with wild type and are presented within a 2 dimensional GNRHR schematic.

The nucleotide sequences for *GNRH1*, *KISS1*, *KISS1R*, *FGFR1*, *PROK2*, *PROK2R*, *KAL1* and *NR0B1* exons and intron-exon boundaries were identical to reference sequences, but we identified two novel missense mutations in the gene encoding the GNRH receptor (*GNRHR1*: NM_000406.2; HGNC: 4421), see Figure 2. The proband’s DNA profile showed two heterozygous mutations, one mutation was a C-to-G transversion at cDNA nucleotide 845 (c.845C>G) leading to an amino acid substitution at residue 282 of proline for arginine (p.282P>R) and the other mutation was A-to-G transition at cDNA nucleotide position 968 (c.968A>G) leading to a substitution at residue 323 of tyrosine for cysteine (p.323Y>C). Subsequently all family members, including the two parents, were screened for *GNRHR1* mutations and the pedigree was determined (Fig. 1). Pro282Arg and Tyr323Cys *GNRHR1* mutations were present in all the proband’s affected brothers but not in his unaffected sister.

The SIFT protein database (sift.jcvi.org) sorts tolerant from intolerant amino acid changes by comparing residues at equivalent positions from different species and assigns a probability of whether a given change is likely to be functional or inactivating. Querying this database with the two novel *GNRHR1* mutations predicted that substitution at both positions, Pro282 and Tyr323, with any other amino acid would be damaging to the receptor function giving both a sift score of 0, where scores <0.05 are predicted to be deleterious.

**Figure 3:**
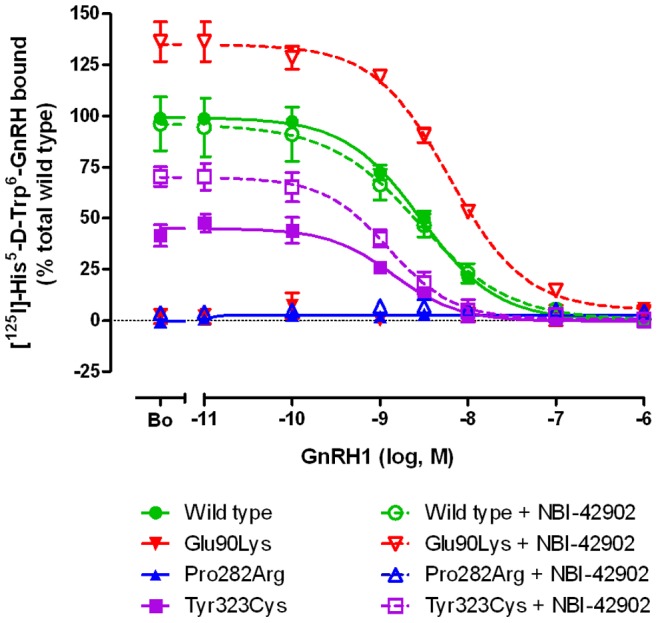
Competitive binding characteristics for Pro282Arg and Tyr323Cys *hGNRHR1* mutants compared to wild type. COS-7 cells were transfected with the expression vector indicated and then grown in the presence of pharmacoperone (NBI-42902) or vehicle (DMSO), washed and then subjected to competitive binding assays using the GnRH1 analog ([^125^I]-His^5^-D-Tyr^6^-GnRH1) and graded concentrations of unlabeled GnRH1. The degree of mutant receptor binding was compared to cells expressing the wild type receptor.

The molecular impact of these two novel GnRHR mutations was tested *in vitro* by expressing each mutant construct in COS-7 cells. When transfected cells were incubated with high concentrations of radiolabeled GnRH1 in the absence of unlabeled ligand, maximal binding to cells expressing Pro282Arg or Tyr323Cys was absent or considerably reduced compared to cells expressing the wild type receptor (Fig. 3 and Table 2). The Tyr323Cys mutant displayed a small increase in binding affinity but maximal radioligand binding was 45±5% of that exhibited by cells expressing the wild type receptor. Pretreatment of cells expressing the Tyr323Cys mutant with NBI-42902 significantly increased maximal radioligand binding to 71±6% of wild type levels but had no effect on cells expressing the Pro282Arg mutant.

**Table 2:** *In vitro* Radioligand Competition Binding Assay.

			+NBI-42902	
	pIC_50_ (nM)	B_max_	pIC_50_ (nM)	B_max_
**Wild type**	8.54±0.04 (2.88)	100±2	8.56±0.17 (2.75)	93±13
**Glu90Lys**	nb	nb	8.21±0.06 (6.17)	134±8*
**Pro282Arg**	nb	nb	nb	nb
**Tyr323Cys**	8.85±0.11 (1.41)	45±5	8.90±0.10 (1.26)	71±6*

Ligand binding affinity (pIC_50_) were measured on intact COS-7 cells transfected with wild type and mutant receptors by competition binding using ^125^I-His^5^-D-Tyr^6^-GnRH1.

*B*_max_ = % of total radioligand bound were expressed relative to wild type receptor without NBI-42902.

nb No measurable binding.

Values are mean ± SEM of three or more independent experiments, converted to nM (in parentheses).

Significant difference of NBI-42902 treatment on *B*_max_ is represented by an asterisk * (p<0.05).

To validate the rescue treatment protocol and to serve as a positive control, the binding and IP responses of the GnRH receptor mutant Glu90Lys was assayed with and without NBI-42902 pretreatment. Previous studies have shown the ability to rescue the maximal binding and IP responses of the Glu90Lys mutant to wild type levels with another cell permeant GnRH antagonist, IN3 [16]. In the absence of NBI-42902 treatment, binding was absent from cells expressing the Glu90Lys mutant but NBI-42902 pretreatment restored maximal binding above wild type levels (134±8%).

**Figure 4:**
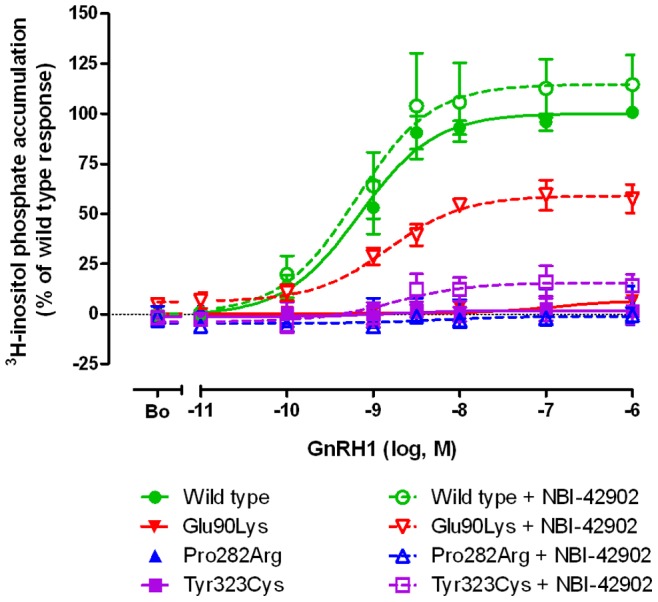
Inositol phosphate accumulation in COS-7 cells expressing wild type *GNRHR1* and the two *hGNRHR1* mutants, Pro282Arg and Tyr323Cys. COS-7 cells were transfected with the expression vector indicated and then grown in the presence of pharmacoperone (NBI-42902) or vehicle (DMSO) and then washed before the addition of myo-[2-^3^H]Inositol. After 16 h the cells were washed again and then incubated for 1 h with graded concentrations of GnRH1. The amount of intracellular accumulation of labeled inositol phosphates in the two mutant expressing COS-7 cells was compared to cells expressing the wild type receptor.

The capacity of each mutant receptor to transduce an intracellular signal by coupling to phosphatidyl inositol hydrolysis was measured by quantifying ligand induced intracellular accumulation of ^3^H-inositol phosphates (IP) (Table 3). In the absence of NBI-42902 pretreatment, both non-rescued mutants lacked the ability to generate significant IP accumulation after incubation of cells with graded concentrations of GnRH1. Even after NBI-42902 pretreatment, the Pro282Arg mutant was unable to generate IP turnover at any GnRH1 concentration whereas this treatment substantially increased maximal IP accumulation to 16±3% of the wild type response in cells expressing the Tyr323Cys mutant (Fig. 4, Table 3). Using NBI-42902 pretreatment, maximal IP response of the Glu90Lys mutant increased to 59±3% of wild type levels.

**Table 3:** *In vitro* [^3^H]-Inositol Phosphate Accumulation Assay.

			+NBI-42902	
	pEC_50_ (nM)	E_max_	pEC_50_ (nM)	E_max_
**Wild type**	9.16±0.12 (0.69)	100±4	9.20±0.23 (0.63)	115±9
**Glu90Lys**	ns	ns	8.82±0.14 (1.51)	59±3*
**Pro282Arg**	ns	ns	ns	ns
**Tyr323Cys**	ns	ns	8.76±0.43 (1.74)	16±3*

IP responses (pEC_50_) were measured in COS-7 cells transfected with wild type and mutant receptors.

*E*_max_ = % of maximal IP responses were expressed relative to wild type receptor without NBI-42902.

ns No measurable stimulation.

Values are mean ± SEM of three or more independent experiments, converted to nM (in parentheses).

Significant difference of NBI-42902 treatment on *E*_max_ is represented by an asterisk * (p<0.05).

**Figure 5:**
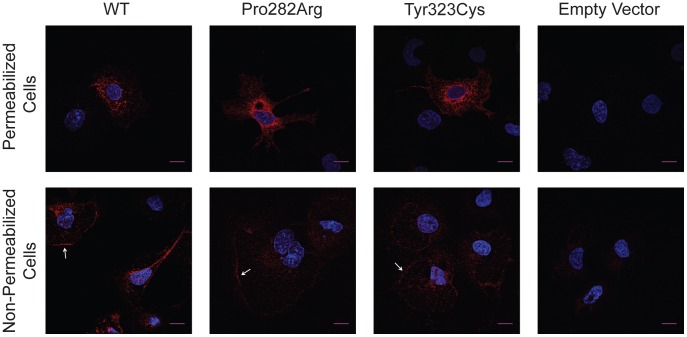
Subcellular localization of heterologously expressed GNRHR1 and mutants, Pro282Arg and Tyr323Cys, in permeabilized and non-permeabilized cells. COS-7 cells were transfected with the expression vector indicated and the immunoreactivity was assessed using indirect methods as described in the methods and materials section. The nuclei are counterstained by Hoechst (blue) and immunofluorescence of the HA-conjugated receptor is shown in red. Z-stack projection of GNRHR distribution in permeabilized (upper panel) and non-permeabilized (lower panel) cells obtained by confocal microscopy. White arrows indicate plasma membrane expression. Scale bar represents 15 µm.

To evaluate the cellular distribution and assess the degree of intracellular retention of the mutant receptors compared to wild type, we performed immunocytochemical studies on permeabilized and non-permeabilized heterologously expressing COS-7 cells. In permeabilized cells Pro282Arg and Tyr323Cys mutants were expressed predominantly in the perinuclear region when compared to wild type receptor (Fig. 5, upper panel), suggesting greater misfolding and cellular retention. In non-permeabilized cells, wild type GnRHR was located at the plasma membrane at a higher proportion than both mutants (Fig. 5, lower panel). Thus these ICC data confirm the substitution of Pro282 by Arginine and Tyr323 by Cysteine impair the proper membrane targeting of the GnRH receptor.

## Discussion

In the family studied here, the male proband was the third son in a kindred of four in which he and his two brothers suffer complete nCHH whilst the sister was unaffected. This observation led us to suspect first an X-linked mode of inheritance of the disease. After excluding mutations in *KAL1* and *NR0B1*, which are associated with X-linked forms of CHH, genes associated with autosomal normosmic CHH or Kallmann syndrome were then screened. We found two novel non-synonymous missense mutations in the gene encoding the GnRH receptor (one mutation affecting each allele). All three affected male patients were compound heterozygous for each mutation whereas the unaffected parents possessed one mutant (either Pro282Arg or Tyr323Cys) and one wild type allele. Whilst the pattern of inheritance appeared X-linked, genetic analysis of this family formally confirmed an autosomal recessive transmission in agreement with previous published cases of GNRHR loss-of-function mutations [4], which confirms that one copy of the wild type *GNRHR1* gene is sufficient for normal gonadotrope function, ruling out haploinsufficiency. In view of the rare but sometimes oligogenic inheritance reported in CHH [17], we screened all other nCHH candidate genes but only found the two *GNRHR1* mutations, suggesting these as the main contributors to the observed nCHH phenotype in this family.

The GNRH receptor is a 328 amino acid tail-less G protein-coupled receptor (GPCR) encoded by a three-exon gene spanning 18.7 kb of chromosome 4q21.2 [2]. The frequency of inactivating mutations in *GNRHR1* is very low with only 22 other natural mutations reported to cause HH identified to date (p.Asn10Lys [18], [19], p.Gln11Lys [20], p.Thr32Ile [5], [19], [21], [22], p.Glu90Lys [5], [19], [23], [24], p.Thr104Ile [25], p.Gln106Arg [11], [19], p.Tyr108Cys [25], p.Ala129Asp [19], [26], Arg139His [18], [19], Arg139Cys [27], p.Pro146Ser [28], p.Ser168Arg [29], p.Ala171Thr [19], [30], p.Cys200Tyr [5], [19], [21], p.Ser217Arg [31], p.Arg262Gln [11], [19], p.Leu266Arg [5], [19], [21], [22], p.Cys279Tyr [5], [19], [21], [22], p.Pro282Arg (described here), p.Tyr284Cys [19], [32], [33], p.Leu314X [19], [34], p.Pro320Leu [20], p.Tyr323Cys (described here) and a splice junction mutation at the intron 1– exon 2 boundary [35]). As these 24 mutations are distributed throughout the *GNRHR1* gene a hotspot for mutations does not seem to exist.

Like other GPCRs, this peptide receptor is folded into seven transmembrane domains (TMDs) linked by alternating intracellular and extracellular loops. Natural inactivating-mutations have contributed to understanding the structure-function relationships necessary for the configuration and stable folding of the receptor, ligand binding, receptor activation and intracellular signal coupling (for review see [2]). Depending on the affected domain, these natural mutations can be classified as either partial-loss or complete loss-of-function. The two novel mutations identified herein (Pro282Arg and Tyr323Cys) apparently disrupt the functioning of TMD6 and TMD7, respectively, via two distinct mechanisms.

The novel Pro282Arg mutation resides in a site in the sixth TMD α-helix in which the proline residue is highly conserved in the rhodopsin family of GPCRs. Pro^6.50^ (superscripts in this form indicate Ballesteros-Weinstein numbering for conserved GPCR residues [36]) is found at the homologous position not only in GnRH receptors from different species (from invertebrates to humans) but is 100% conserved in family A GPCRs [37]. The crystal structure of rhodopsin and other model GPCRs have revealed that TMD6 is kinked due to this conserved proline [38], [39]. Proline residues weaken transmembrane α-helices and kink protein structures due to irregular backbone hydrogen bonding and the presence of a bulky pyrrolidine ring. In rhodopsin and other family A GPCRs Pro^6.50^ is thought to act as a pivot around a cluster of flexible aromatic residues (Phe^6.44^, Trp^6.48^, Tyr^6.51^ and Tyr^6.52^ in the human GNRHR1). In this model, toggling of Trp^6.48^ modulates the bend angle of TMD6 around Pro^6.50^ so that receptor activation transduces large conformational changes at the cytoplasmic interface of TMD6 [37], [38], [40]. Modeling the wild type human GNRHR1 in the active conformational state confirmed that disrupted intramolecular interactions involving TMD6 (Cys^6.47^ and Asn^7.45^, and between Met^3.43^ and Phe^6.40^) led to a small clockwise rotation (viewed from the extracellular side) of the intracellular portion of TMD6 around the Pro^6.50^ hinge [41]. In addition to being central to the toggle-hinge mechanism, Pro^6^.^50^ is neighboring a hydrophobic pocket formed by Trp^6.48(280)^, Tyr^6.51(283)^, Tyr^6.52(284)^, Phe^5.43(216)^ and Tyr^3.37(126)^ [42] and substitution to arginine would not only drastically disrupt the proline-induced structural constraints but would also change the native charge environment due to the basic arginine side chain.

The Pro282Arg mutation is the third amino acid mutation to be found in TMD6 of GNRHR, but is structurally and functionally the most severe. The complete lack of both binding and inositol phosphate accumulation even when treated with a receptor trafficking-rescue regimen can be explained by destruction of the global receptor structure and/or ligand binding pocket, and/or failure to be targeted efficiently to the plasma membrane. Our immunocytochemical analysis detected mutated Pro282Arg receptor protein throughout the cell and some at the cell membrane (Fig. 5) indicating that a proportion of receptor is able to escape ER quality control mechanisms. This suggests that disruption of the active receptor conformation and/or decreased ligand binding pocket is the principal cause for the complete-loss-of-function of this mutant receptor. The other previously described TMD6 mutants (Cys279Tyr and Tyr284Cys) have either absent binding and IP activation [22] or decreased binding and IP activation, respectively [32], but unlike Pro282Arg, their function was rescued to some extent after pharmacoperone (IN3) treatment [5], [19], [22].

The second novel mutation identified in our patients (Tyr323Cys) resides towards the intracellular portion of TMD7 within the highly conserved N/DPxxY motif. Movements essential for receptor activation promote the interaction of Tyr323 with the side chain of Arg^3.50^ (TMD3) [43], [44]. This interaction stabilizes the active receptor conformation and mediates ligand-induced signal transduction [45], [46]. The essential role of Tyr323 in G protein-signal transduction has been evidenced by mutation of Tyr323 to alanine which increased receptor expression 2.5-fold compared to wild type but resulted in poor Gα_q/11_ coupling [45], [46]. Interestingly, substitution of Tyr323 to Phe, which preserves the aromatic nature of the residue but eliminates the side chain hydroxyl-group resulted in normal G protein-activation as assayed by induced inositol phosphate accumulation [45]. Our results corroborate the importance of maintaining this aromatic side chain for active G protein signaling as the Tyr323Cys mutation, which substitutes the aromatic side chain with one that is hydrophobic and polar, was able to bind radioligand but completely lacked ligand induced inositol phosphate accumulation. After treatment with NBI-42902, where plasma membrane expression of the mutant receptor was increased to 71% of wild type levels, only small levels of IP accumulation was detected (16% of wild type levels) indicating the impairment of G protein-activation is compounded by ineffective plasma membrane routing.

When compared to the other naturally occurring TMD7 mutants, the Tyr323Cys mutation is the least severe when assayed *in vitro*. Both, Leu314X, a nonsense truncation mutation, and Pro320Leu, a missense mutation, lacked GnRH binding and production of inositol phosphates in response to GnRH stimulation [20], [47].

The hormonal profiles of our three affected patients harboring the Pro282Arg/Tyr323Cys compound mutations support the findings that these mutated GnRH receptors lack residual biological activity and present with complete CHH. In two of the affected males there was absent gonadotropin response after GnRH stimulation. The clinical phenotype of our patients mirrored the severity of their hormonal deficiency with small prepubertal testicular volume and underdeveloped external genitalia. Micropenis and small intrascrotal testes, sometimes cryptorchid, are reported in male patients with *GNRHR1* mutations [48], likely reflecting insufficient secretion of gonadotropins and testosterone during the prenatal period.

In conclusion, we have identified two novel GNRHR gene mutations present in members of a family of French Caucasian origin. The severe phenotypic features of complete normosmic congenital hypogonadotropic hypogonadism in compound heterozygous patients demonstrate the deleterious effect of both mutations, one of which can be classified as a partial and the other a full loss-of-function mutation *in vitro*.
